# Selection on a small genomic region underpins differentiation in multiple color traits between two warbler species

**DOI:** 10.1002/evl3.198

**Published:** 2020-10-19

**Authors:** Silu Wang, Sievert Rohwer, Devin R. de Zwaan, David P. L. Toews, Irby J. Lovette, Jacqueline Mackenzie, Darren Irwin

**Affiliations:** ^1^ Department of Zoology and Biodiversity Research Centre University of British Columbia Vancouver BC V6T1Z4 Canada; ^2^ Department of Biology and Burke Museum University of Washington Seattle Washington 98195; ^3^ Department of Forest and Conservation Sciences University of British Columbia Vancouver BC V6T1Z4 Canada; ^4^ Department of Biology 619 Mueller Laboratory Pennsylvania State University University Park Pennsylvania 16802; ^5^ Fuller Evolutionary Biology Program Cornell Lab of Ornithology Ithaca New York 14850

**Keywords:** ASIP, cline, GWAS, hybrid zone, hybridization, pigmentation, RALY, *Setophaga*, speciation

## Abstract

Speciation is one of the most important processes in biology, yet the study of the genomic changes underlying this process is in its infancy. North American warbler species *Setophaga townsendi* and *Setophaga occidentalis* hybridize in a stable hybrid zone, following a period of geographic separation. Genomic differentiation accumulated during geographic isolation can be homogenized by introgression at secondary contact, whereas genetic regions that cause low hybrid fitness can be shielded from such introgression. Here, we examined the genomic underpinning of speciation by investigating (1) the genetic basis of divergent pigmentation traits between species, (2) variation in differentiation across the genome, and (3) the evidence for selection maintaining differentiation in the pigmentation genes. Using tens of thousands of single nucleotide polymorphisms (SNPs) genotyped in hundreds of individuals within and near the hybrid zone, genome‐wide association mapping revealed a single SNP associated with cheek, crown, breast coloration, and flank streaking, reflecting pleiotropy (one gene affecting multiple traits) or close physical linkage of different genes affecting different traits. This SNP is within an intron of the RALY gene, hence we refer to it as the RALY SNP. We then examined between‐species genomic differentiation, using both genotyping‐by‐sequencing and whole genome sequencing. We found that the RALY SNP is within one of the highest peaks of differentiation, which contains three genes known to influence pigmentation: ASIP, EIF2S2, and RALY (the ASIP‐RALY gene block). Heterozygotes at this gene block are likely of reduced fitness, as the geographic cline of the RALY SNP has been narrow over two decades. Together, these results reflect at least one barrier to gene flow within this narrow (∼200 kb) genomic region that modulates plumage difference between species. Despite extensive gene flow between species across the genome, this study provides evidence that selection on a phenotype‐associated genomic region maintains a stable species boundary.

**Impact Summary**

Speciation is a very important process generating the diversity of lifeforms. However, study of the genomic basis underlying speciation is still in its infancy. Here, we took advantage of a natural hybrid zone (where diverged/diverging lineages interbreed) between two wood warbler species in the Pacific Northwest to uncover the genomic basis underlying species‐specific plumage variation, which is known to be used in social signaling within and between species. We found a 0.2‐Mb gene block, including three pigmentation genes ASIP, EIF2S2, and RALY, significantly explains variation in four of the seven plumage traits that differ between species. This region is also one of the most differentiated peaks in the genome, whereas the rest of the genome is mostly similar between species. Further tracking this gene block across the hybrid zone over two decades revealed selection against hybrids within this gene block that could be one of the predominant evolutionary forces maintaining the species boundary between these hybridizing sister species.

Speciation involving partial geographic isolation commonly occurs in nature, yet we are just beginning to understand the genomic changes underlying this process (Nosil et al. 2009; Nosil and Feder 2012; Payseur and Rieseberg 2016; Campbell et al. 2018). Differentiation in allopatry is often followed by a period of expansion and population contact, when differentiated forms may then partially hybridize but not blend completely back into a single species (Wu 2001; Weir and Schluter 2004). In such cases, stable hybrid zones provide opportunities to examine which parts of the genome show strong and stable differences between species, as well as which parts are associated with traits that distinguish species (Slatkin 1973; Barton and Hewitt 1989; Barton 2001; Buerkle and Lexer 2008).

During differentiation in allopatry, mutation and genetic drift cause differentiation throughout the genome (Mayr 1942; Coyne and Orr 2004; Feder et al. 2013), whereas positive selection is expected to cause reductions in diversity at specific loci and those regions closely linked to them, driving up relative differentiation between the populations in those regions (Hartl 1977; Wu 2001; Via 2009; Nosil and Feder 2012). Upon secondary contact, hybridization and backcrossing can result in gene flow and homogenization of the two populations across the genome. At some regions, suboptimal combinations of alleles can cause reduced fitness of hybrids and backcrosses, diminishing introgression. The resulting “islands of differentiation” can gradually expand over time (Wu 2001; Bradshaw and Schemske 2003; Turner et al. 2005; Nosil et al. 2009; Schluter and Conte 2009; Via 2009) due to physical linkage and mutation in nearby genomic regions, a phenomenon known as divergence hitchhiking (Nosil et al. 2009; Via, 2009, 2012; Feder et al. 2012). An important question about these processes is whether the key differences between the species are underlain by a few genomic regions of major effect or many regions of smaller effect spread widely through the genome.

Divergent traits, for example pigmentation differences, can cause hybrids to have lower fitness (Servedio and Noor 2003; Bridle et al. 2006) and/or contribute to social interactions related to mate choice and/or competitive interactions (West‐Eberhard 1984; Sætre et al. 1997; Hill and Mcgraw 2004; Anderson and Grether 2010). Carotenoid and melanin pigmentation are often involved in species‐diagnostic traits (Sætre et al. 1997; Mikami et al. 2004; Seehausen and Schluter 2004; Kronforst et al. 2006; Uy et al. 2009; Hill 2015; Toews et al. 2016a,b; Barrera‐Guzmán et al. 2018). Within birds, a group that has played a prominent role in the speciation literature (Mayr 1942, 1970; Coyne and Orr 2004; Price 2007), the patterning of colors often differs between species and these color patterns are often involved in mate choice favoring conspecifics (Sætre et al. 1997; Uy et al. 2009; Hill 2015). In such cases, the regulatory genes for melanin and carotenoid pathways tend to differentiate between species as they contribute to species‐specific plumage patterns (Hoekstra 2006; Massey and Wittkopp 2016). Although genetic regions underlying divergent pigmentations have been identified among some recently diverged lineages (Uy et al. 2009; Poelstra 2013; Toews et al. 2016a,b; Barrera‐Guzmán et al. 2018; Knief et al. 2019), how these pigmentation genes contribute to speciation in the face of gene flow remains elusive. In theory, sexual and social selection in allopatry can result in low fitness in hybrids, due to genetic incompatibilities and/or rare‐mating‐type disadvantage (Irwin 2020).

Secondary contact hybrid zones, locations where previously geographically isolated lineages have come into contact and now interbreed, provide opportunities to understand the relationship between species‐diagnostic features and genomic differentiation as well as the evolutionary forces shaping such genomic regions. The recombination that occurs in extensive hybrid zones allows detection of associations between phenotypic traits and genotypes at specific loci. There are now well‐developed methods to conduct such genome‐wide association studies (GWAS) while controlling for overall genomic differentiation between species (Buerkle and Lexer 2008; Shriner 2013). Furthermore, the strength of selection maintaining species differences can be inferred from the width of the cline (i.e., the transition zone) in the genotype or phenotype of interest (Barton and Gale 1993; Nurnberger et al. 1995).

Here, we examine a hybrid zone (Fig. 1A) between *Setophaga townsendi* (abbreviated as *townsendi*; distributed in Northwest United States and Western Canada) and *Setophaga occidentalis* (abbreviated as *occidentalis*; distributed along the west coast of the contiguous United States) along the Cascade mountains in North America. These closely related species of wood‐warblers started diverging an estimated 400,000 years ago (Rohwer and Wood 1998; Pearson and Rohwer 2000; Weir and Schluter 2004; Krosby and Rohwer, 2009, 2010). Based on inferences from mitochondrial DNA, the two species have most likely been geographically separated during long periods of Pleistocene glaciation and were inferred to come into secondary contact within the past 12,000 years, following the last major glacial cycle (Krosby and Rohwer 2009).

**Figure 1:**
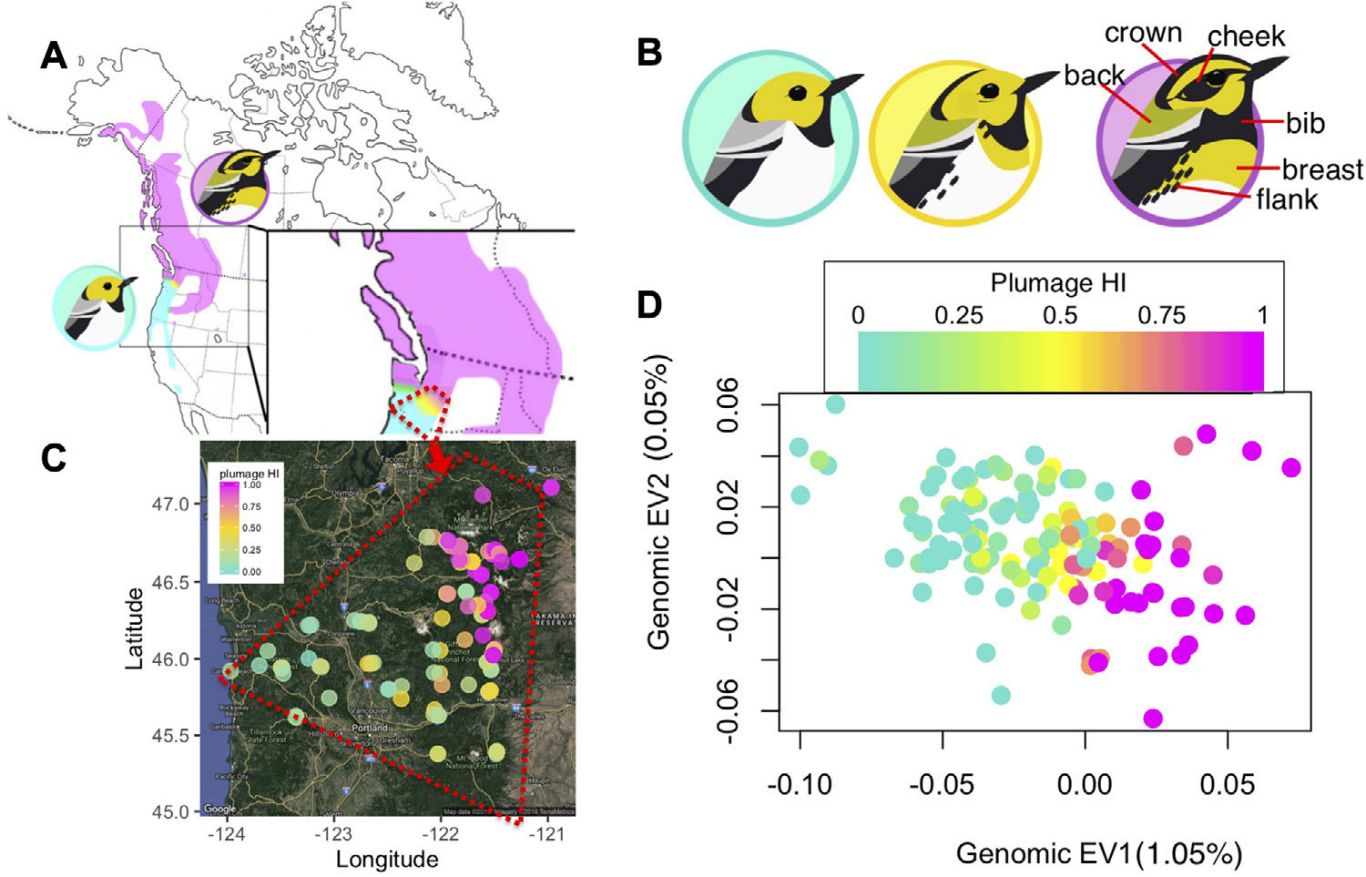
**Plumage variation across the hybrid zone is consistent with genomic variation. A**, Range map of *Setophaga occidentalis* (turquoise) and *S. townsendi* (magenta) with the cascade hybrid zone colored in yellow. **B**, Illustration highlighting the key plumage difference between *Setophaga occidentalis* (left), hybrids (center), and *S. townsendi* (right) (illustration by Gil Jorge Barros Henriques). **C**, A map showing site mean plumage hybrid index based on eight plumage traits (0 for pure *occidentalis*, in turquoise; 1 for pure *townsendi*, in magenta) near the hybrid zone. **D**, Genomic eigenvector 1 (EV1, explaining 1.05% of the total variation) and eigenvector 2 (EV2, explaining 0.05% of the total variation) of the principal components analysis of genomic covariance, with individual data points colored by plumage hybrid index. The genomic EV1 reflects the variation among individuals that are *occidentalis*‐like (low EV1) versus *townsendi*‐like (high EV1).

Males of the two species differ in several plumage features related to both carotenoid and melanin patterning on the crown, cheek, breast, flank, and back. For example, *townsendi* has a melanin‐based cheek patch (Fig. 1B, right), whereas *occidentalis* displays a completely carotenoid‐based cheek (Fig. 1B, left). Because apparent hybrids predominantly resemble *occidentalis* in crown and cheek coloration (Fig. 1B center), with intermediate breast (the extent of yellow plumage) and back colors (green plumage in the mantle), Rohwer and Wood (1998) inferred that face coloration of *occidentalis* and hybrids would be controlled by a single‐locus dominant allele. These signals can be important in male‐male competition for territories (Pearson 2000; Pearson and Rohwer 2000), and likely play a role in mate choice as well.

We used GWAS to investigate the genomic basis for variation in seven color pattern traits that differ between *townsendi* and *occidentalis*, using tens of thousands of single nucleotide polymorphisms (SNPs) in hundreds of individuals from the hybrid zone. We also examined patterns of genomic differentiation between these species, using a smaller set of individuals far from the zone. We asked the following specific questions: (1) Are there individual SNPs and/or islands of differentiation strongly associated with species‐specific features (crown, cheek, bib darkening, breast yellow, flank streaking, and greenish back); (2) Is cheek darkening influenced largely by an allele of dominant effect, consistent with the inference made two decades ago by Rohwer and Wood (1998), and what are the inheritance patterns of the other traits; and (3) Is the locus‐specific cline narrower than the genomic cline, and are widths similar between time periods, consistent with stable selection maintaining genetic differentiation underlying the phenotypic differences between the species?

## Materials and Methods

### SAMPLING

A total of 265 adult birds are involved in this study (see Table S1 summarizing the sampling counts), which includes historical specimens at the Burke Museum of Natural History and Culture (University of Washington, Seattle, WA) collected in 1987–1994 (Rohwer and Wood 1998); and 2005–2008 (Krosby and Rohwer 2010); as well as resampling of similar sites in 2015–2016 (Fig. 1C) across the hybrid zone and extending into the ranges of *townsendi* and *occidentalis*. Among the 265 individuals (Table S1), 250 of them were involved in our previous work in this system (Wang et al. 2019) with 15 additional samples from 2005–2008 sampling included in this study to increase the sample size.

### PLUMAGE MEASUREMENTS

Melanin‐ and carotenoid‐based plumage traits allow identification of the two species (Fig. 1B), and there is also some variation within each species (Rohwer and Wood 1998; Owen‐Ashley and Butler 2004). To quantify plumage variation within and between populations, we focused on seven distinct plumage traits in males: (1) cheek yellow versus black coloration, (2) crown yellow versus black coloration, (3) throat bib darkening, (4) intensity and (5) extent of breast yellow, (6) presence of black streaks on the flank, as well as (7) intensity of green chroma on the back (Fig. 1B).

All 265 warblers were photographed by SW, with FinePix HS50EXR (Fujifilm, Fuji, Japan) in automatic mode for plumage analysis. We took three pictures from different angles: (1) frontal with head tilted up (for bib and breast measurements), (2) profile (cheek, flank), and (3) from above (crown) (Fig. S1). Instead of scoring species‐specific plumage variation as discrete variables (Wang et al. 2019), for the present study we further quantified pigmentation of the patches using continuous variables. A single observer (JM) processed all field and museum photos (Table S1). First, the photos were quality filtered to ensure visibility of each plumage variable, resulting in reduced sample sizes summarized in Table S2. Plumage color metrics were measured using Adobe Photoshop CC in CIE (*Comission Internationale de l'Eclairage*) LAB color space. LAB color space is a three‐dimensional space consisting of three distinct, perpendicular axes: (1) Luminosity (L) ranging from black (0) to white (100), (2) “A” ranging from green (–100) to red (100), and (3) “B” ranging from blue (–100) to yellow (+100; Adobe 2017). We chose this color space because it linearizes the variables of interest along three distinct axes: black (“L”), green (“A”), and yellow (“B”).

For the crown, cheek, and breast, we selected a standardized area (see below) and averaged the pixels to record the values, respectively. The intensity of bib black was captured in the “L” axis. The intensity of green coloration on the back was captured by the “A” axis. The coloration on crown, cheek, and breast was captured by the “B” axis. For the cheek, we selected and averaged the entire area from above the eye (but excluding the eye) to the bib, and from the base of the bill to the back. To measure the extent of yellow on the breast, we used the program Analyzing Digital Images (ADI; Snyder 2011). We quantified the length from the lower edge of the bib toward the lower edge of the yellow breast. The lower and mid flank streaking were scored in our previous study (Wang et al. 2019), and we averaged the lower and mid flank streaking scores to reflect overall flank streaking.

Differences in ambient light conditions at the time a picture is taken can confound comparison of color metrics among individuals. To address this, we used the white balance feature in Photoshop, using the white plumage of each individual's belly as a standard, to correct for differences in ambient light among photos and standardize the color metrics. This way, we estimated the relative pigments within each individual. This is appropriate because the relative intensity of pigment compared to the background (rather than the absolute pigmentation) is important for social signaling (Endler 1992). Ideally, we could include a white balance in addition though we did not. However, based on extensive observation with the birds in the field and museum collection, we are confident that there is little variation in the white plumage. To control for age effects on black and yellow pigmentation, age corrections of the seven color variables were done following our previous study (Methods in Supporting Information) (Wang et al. 2019). Because a small subset of the samples were museum specimens, which experience color decay over the course of preservation, we subsequently tested for such an effect and performed color correction to control for it (detailed in Methods in Supporting Information) before GWAS.

We acknowledge that without spectral analysis, we do not incorporate UV reflectance, which is a common aspect of signaling in avian systems (Eaton and Lanyon 2003). However, our methods do allow us to estimate the relative intensity of melanin‐ and carotenoid‐based plumage traits during the breeding season, as neither of them is highly associated with UV reflectance.

### GENOTYPING‐BY‐SEQUENCING DATA

The GBS data (aligned to *Taeniopygia guttata* reference version 3.2.4 [Warren et al. 2010]) of 265 individuals were partly acquired (*N* = 250) from our previous study (Wang et al. 2019), with data from 15 additional samples included here (Table S1). In the current study, we further analyzed the GBS data in depth, in terms of detecting the genetic basis of divergent plumage patches and examining genetic differentiation chromosome‐by‐chromosome. For details of the GBS analysis, please see Methods in Supporting Information.

### GENOME‐WIDE ASSOCIATION STUDIES

To identify SNPs that are associated with variation in the five plumage traits, we conducted GWAS with the GenABEL package in R (Aulchenko et al. 2007) using the GBS and plumage data. The GenABEL function *ibs*, which calculates identity‐by‐state relatedness among individuals, was used to control for population structure. Phenotype‐genotype association was conducted with the *egscore* function. Genomic control of inflation factor *λ* (which represents the effect of genetic structure and sample size; Aulchenko et al. 2007) was estimated (with the *estlambda* function) from the genomic data assuming that randomly selected markers from the genome are not associated with the trait (after controlling for population structure). We then used *λ* to correct the test statistic *χ* ^2^ of each association test, so that the test is relative to the null hypothesis of no phenotype‐genotype association (Aulchenko et al. 2007).

To find the number of independent hypotheses for multiple hypothesis correction, we applied a significance threshold of alpha = 10^−5^ to determine candidate SNPs, as a balance between full Bonferroni correction (0.05/21,852 SNPs = 2.3 × 10^−6^) and the lack of independence among sets of SNPs due to linkage. Genes associated with the candidate SNPs (i.e., those with *P*‐values below this alpha value) were retrieved from Ensembl Zebra Finch (*Taeniopygia guttata*) (taeGut 3.2.4 [Zerbino et al. 2018]).

### WHOLE GENOME SEQUENCING

To sample genetic variation at a greater resolution (more SNPs per genetic region) along chromosomes compared to the GBS data, we selected five samples from *occidentalis* in Pinehurst, CA (UWBM 66152, 66153, 66148–66150) and five samples from inland *townsendi* in Tok, AK (UWBM 84816–84819, 84860) for whole genome resequencing. For DNA extraction, we used 2 mm^3^ of tissue digested in buffer ATL and AL and washed with AW1 and AW2 of the Qiagen DNeasy Blood and Tissue kit—following the tissue extraction procedure—and separated DNA using UPrep spin columns (Genesee). We standardized DNA concentrations after quantifying concentrations with a Qubit fluorometer, and then generated sequencing libraries with the Illumina TruSeq Nano kit—which includes an eight‐cycle PCR enrichment—selecting 350 bp insert sizes. We individually indexed each sample and sequenced the combined libraries across a single lane of an Illumina NextSeq 500 using the paired‐end 150 bp sequencing chemistry. We combined these 10 samples with other wood warblers from other projects, and consistently included 24 individuals per sequencing lane to generate the target depth of 4–5× across samples.

### GENOMIC DIFFERENTIATION

To quantify the level of differentiation throughout the genome between species using the GBS data, which has higher depth of coverage and sample size than the whole genome sequencing (WGS) data, we calculated *F*_ST_ (Weir and Cockerham 1984) with VCFtools 0.1.14 (Danecek et al. 2011) between allopatric *townsendi* (Montana and Idaho; *N* = 38) and *occidentalis* (Oregon and California; *N* = 23). This was done for each of the filtered SNPs, and then a genome‐wide weighted average was calculated according to Weir and Cockerham (1984). We focus on *F*_ST_, rather than absolute nucleotide distance *D_xy_*, because Matthey‐Doret and Whitlock (2019) have shown that background selection can affect *D_xy_* but has little effect on *F*_ST_ for a young species pair.

The genomic landscape of differentiation based on the GBS data was compared to that based on the WGS data. The reads from the WGS data were aligned to the same Zebra Finch reference (version 3.2.4) (Warren et al. 2010) with bwa (Li and Durbin 2010) using default settings. ANGSD (Korneliussen et al. 2014) version 0.930/0.931 was used to account for genotyping uncertainty using low‐depth WGS data before estimating *F*_ST_ (with ANGSD) for each of the nonoverlapping 10‐kb windows.

### DETECTING SELECTION IN THE RALY REGION

As one SNP within the RALY gene (see Results) was significantly associated with multiple plumage landmarks, we investigated whether this locus shows a signature of selection (i.e., low hybrid fitness) by tracking the spatial and temporal variation in this locus relative to the plumage hybrid index and rest of the genome (using samples described previously [Wang et al. 2019]). We fit the relationship between geographic location and RALY SNP allele frequencies using an equilibrium geographic cline model (Szymura and Barton 1986; Gay et al. 2008).

To infer whether selection has recently shaped the spatiotemporal distribution of alleles of the RALY SNP, we used a geographical cline method we developed (Wang et al. 2019). This method (see Methods in Supporting Information) uses sampling across multiple time periods to test whether the increase in the widths of clines (represented by *w* ^2^_2015‐2016_ – *w* ^2^_1987‐1994_) is less than expected under neutral diffusion. In the present study, we applied this method to the RALY clines. In addition, we included the genomic clines and plumage clines (of 1987–1994 and 2015–2016) from our previous study (Wang et al. 2019). The RALY cline was calculated based on the same set of samples as the genomic cline. We calculated 95% bootstrap confidence interval of *w* ^2^_2015‐2016_ – *w* ^2^_1987‐1994_, by resampling sites within each sampling period as detailed in Wang et al. (2019). If the selection is acting more directly at the RALY region, the width of RALY cline should be less than the genome‐wide clines, and the change of the RALY cline width over decades should be less than that of the genome‐wide cline and that predicted under neutral diffusion.

## Results

### GENOMIC BASIS OF PLUMAGE COLORS

**Figure 2:**
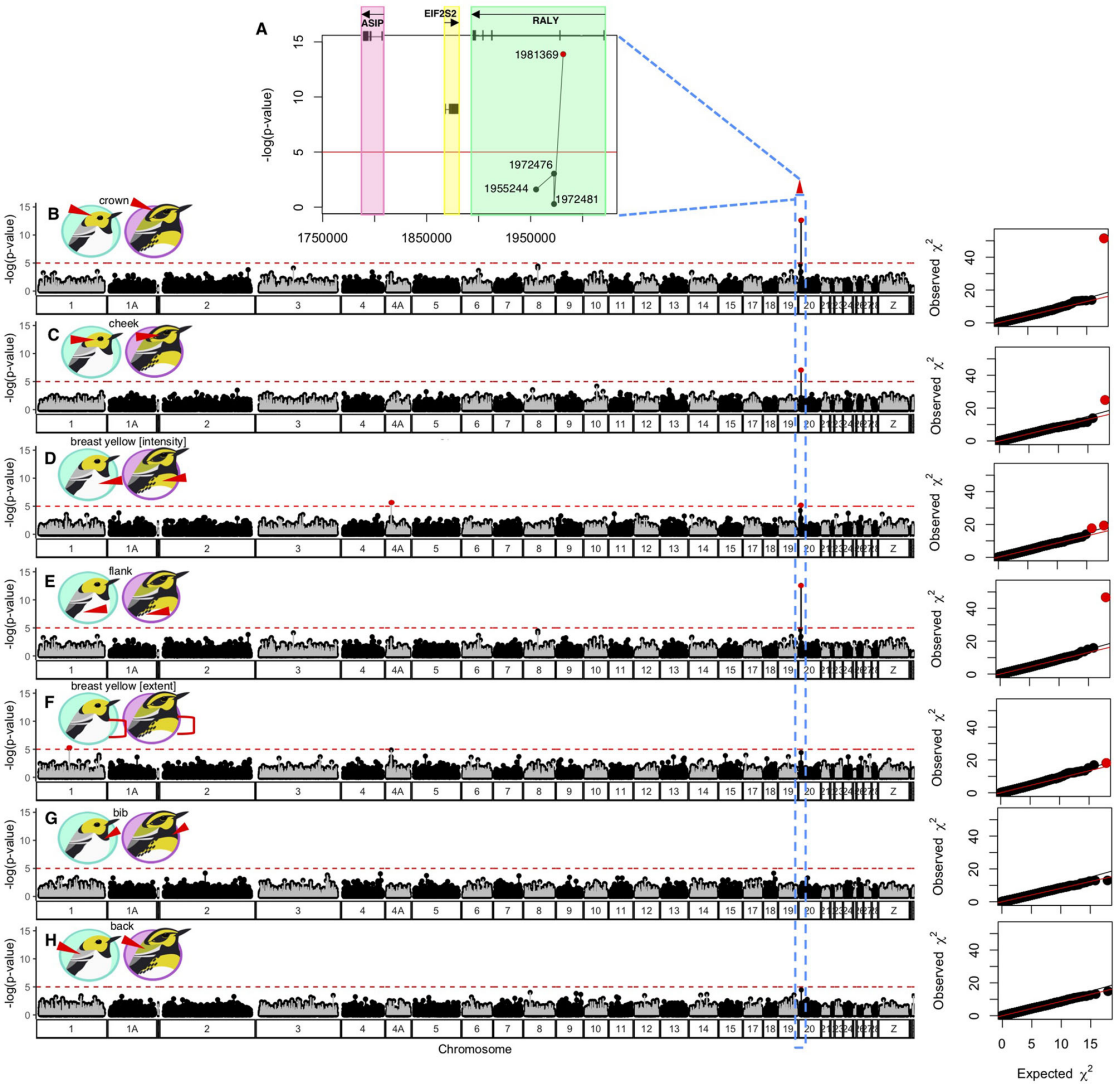
**GWAS revealed a SNP within ASIP‐RALY gene block associated with multiple traits**. This SNP is inside the RALY gene in chromosome 20 at position 1981369 that was significantly associated with crown (**A** and **B**), cheek darkening (**C**), and flank streaking (**E**). Results are also shown for the intensity (D) and extent of breast yellow (**F**), bib darkening (**G**), and the color of the back (**H**). **A–H**, genomic scans of inflation factor‐corrected –log (*P*‐value) of genotype‐phenotype association tests. The horizontal red lines represent the critical threshold (10^−5^). The plumage trait tested in each scan is indicated by the red triangle in the cartoons. For each phenotype, the *χ* ^2^ plot on the right shows the deviation of observed *χ* ^2^ of each SNP away from the expected *χ* ^2^ value under the null model in which the SNP has similar effect size on the trait as the rest of the genome. In **B**, **C**, **D**, and **E**, there is a strong peak at chromosome 20 position 1981369 (red dot) inside the RALY gene that indicates an association with crown, cheek, breast coloration, and flank streaking. **A**, Genetic map of the ASIP‐RALY gene block on chromosome 20 and all available SNPs in that region in the GBS dataset. The colored boxes represent the stretches of the protein‐coding genes, ASIP (red), EIF2S2 (yellow), and RALY (green), with the arrows indicating whether the gene is located on the reverse (pointing to the left) and forward (pointing to the right) strand; exons are depicted as the black vertical bars connected by introns (black horizontal lines).

**Figure 3:**
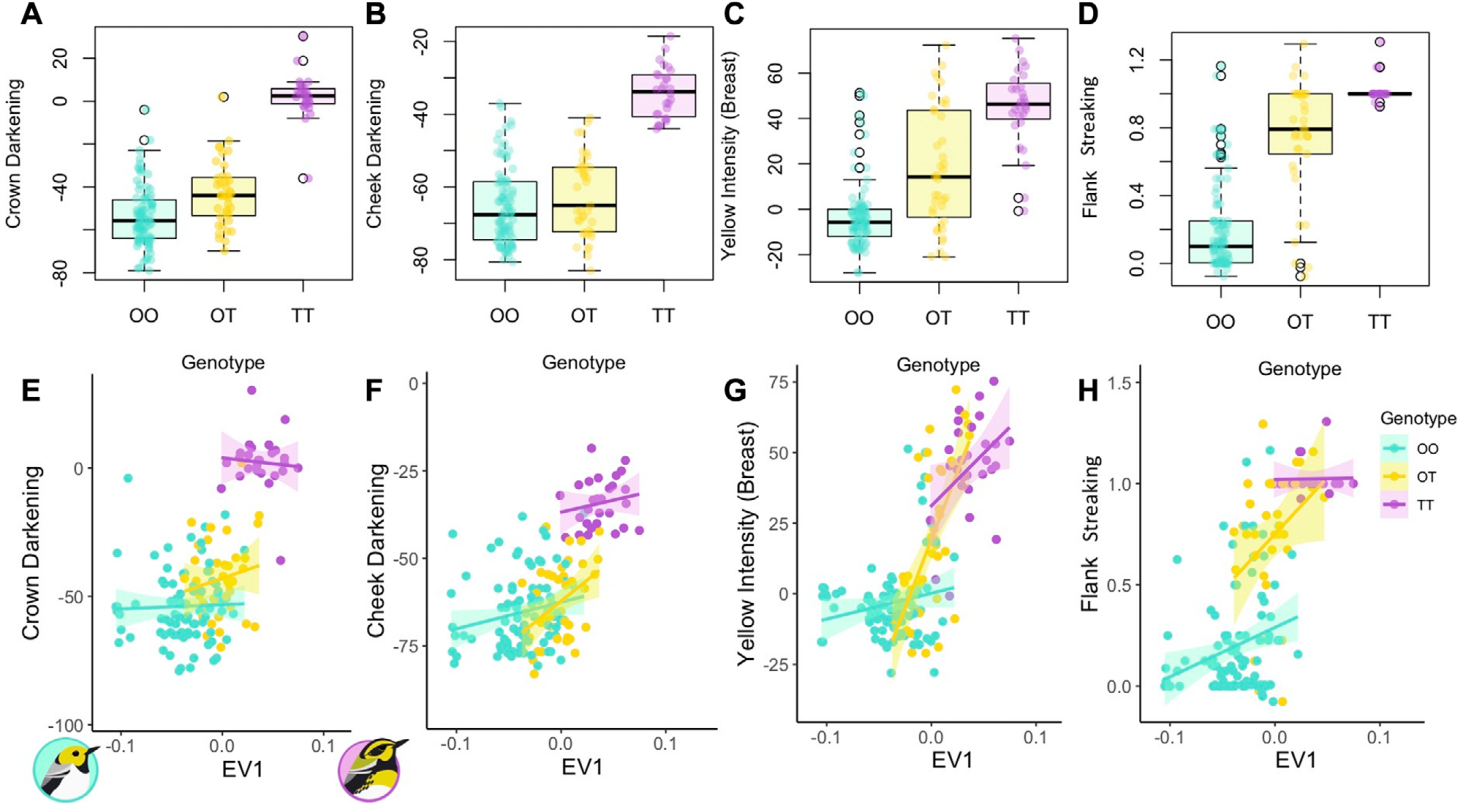
**RALY SNP‐phenotype association**. Association of the RALY SNP with crown (**A** and **E**), cheek (**B** and **F**), breast coloration (**C** and **G**), as well as flank streaking (**D** and **H**), in which the pure *occidentalis* genotypes is denoted as “OO,” pure *townsendi* genotype is denoted as “TT,” and heterozygotes as “OT.” Each small symbol represents the phenotype of a single individual, either grouped by genotype alone (**A–D**) or graphed by underlying genomic ancestry and colored by genotype (**E–H**). This figure shows only the phenotypic traits that showed significant (*P* < 10^−5^) associations with the RALY SNP after accounting for the underlying genomic ancestry. The data are consistent with partial dominance of the O allele on crown, cheek, and breast coloration, but partially recessive effect of the O allele on flank streaking.

**Figure 4:**
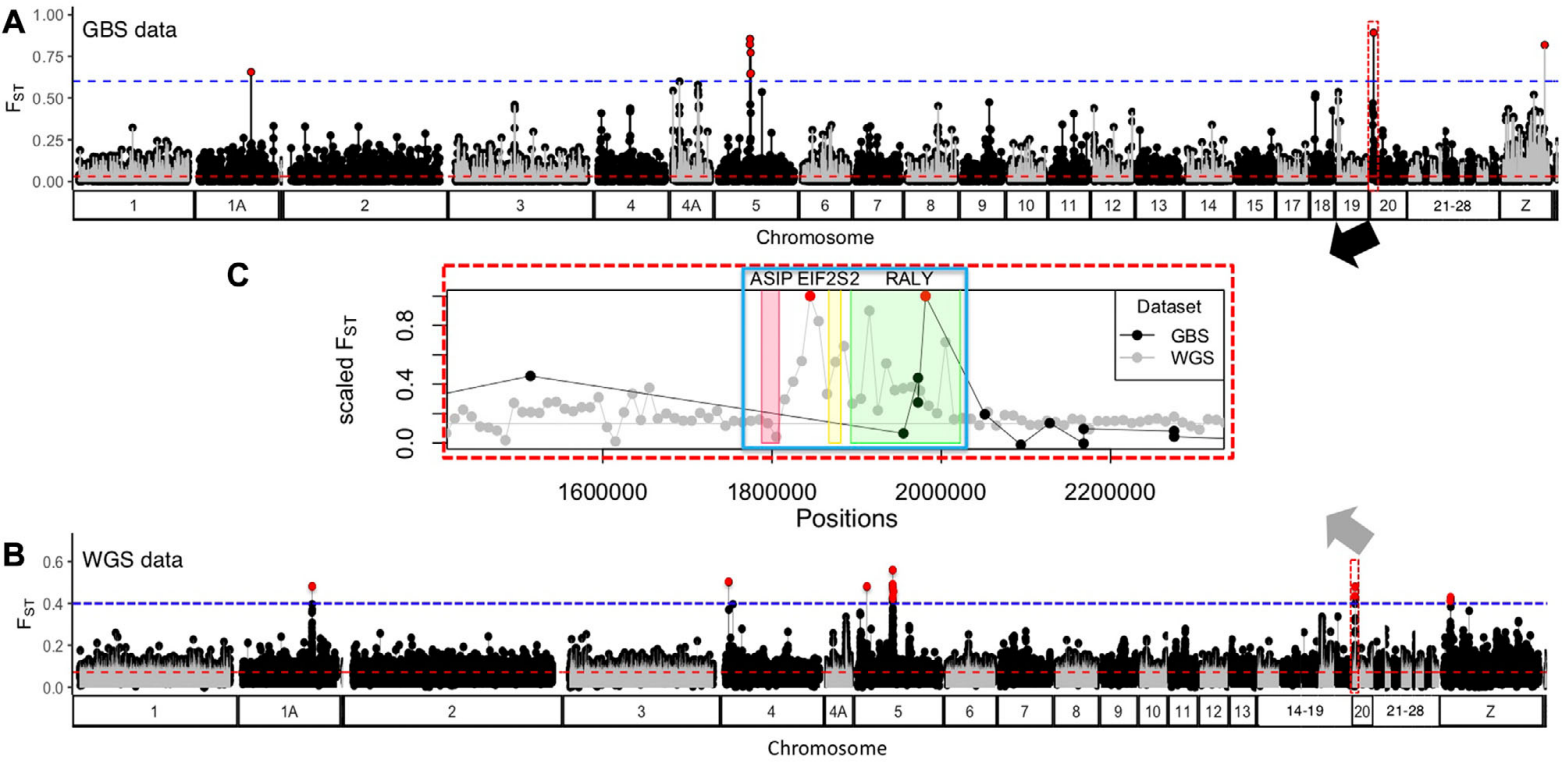
**Genomic variation in differentiation between inland *townsendi* and *occidentalis***. Variation in *F*_ST_ as estimated per‐SNP using the GBS data (**A**) or averaged per genomic window using the WGS data (**B**). **C**, Scaled *F*_ST_ (relative to the maximum *F*_ST_ of chr20) along the ASIP‐RALY gene block (in the blue border, with the genetic range of ASIP colored in red, EIF2S2 in yellow, and RALY in green) and its flanking region on chr20. The GBS data points are black, whereas the WGS data points are gray (**C**). The GBS data (**A**) show a few regions of high differentiation, whereas the rest of the genome is similar between species (Weir and Cockerham weighted average *F*_ST_ of 0.03 is indicated by the horizontal red dotted line). The outliers (>0.6, blue dotted line) are colored in red. Consistent with the GBS data, window‐based *F*_ST_ estimated using the WGS data (**B**, genome‐wide *F*_ST_ of 0.08, indicated by the horizontal dotted red line) also revealed strong *F*_ST_ peaks (>0.4, indicated by the red dots), including within the ASIP‐RALY gene block (**B** and **C**). The lengths of chromosomes between GBS (**A**) and WGS (**B**) are different because GBS data sampled uneven subsets of different chromosomes.

Across the Cascade mountain range, the *occidentalis* plumage type was observed to the southwest and the *townsendi* plumage type was to the northeast (Rohwer and Wood 1998; Wang et al. 2019) (Figs. 1A and 1C). The overall pattern of genomic differentiation was consistent with the plumage differentiation: principal components analysis of the genomic covariance resulted in a first axis (eigenvector 1) that qualitatively distinguishes *occidentalis* and *townsendi* plumage types (Fig. 1D). GWAS revealed that a single SNP is strongly associated with the colors of the crown, cheek, and flank (Fig. 2; crown: *χ* ^2^ = 51.54, *P* = 1.30 × 10^−14^, Figs. 2A and 2B; cheek: *χ* ^2^ = 25.85, *P* = 4.72 × 10^−8^, Fig. 2C; flank: *χ* ^2^ = 44.11, *P* = 1.55 × 10^−12^, Fig. 2E). Although the intensity of breast color was not significantly associated with this SNP at our chosen alpha value of 10^−5^, it was close (Fig. 2D; breast color intensity: *χ* ^2^ = 17.56, *P* = 2.78 × 10^−5^; Fig. 3C). This SNP maps to chromosome (chr) 20 of the reference genome and is within an intron of the gene RALY (Fig. 2A), which encodes heterogeneous nuclear ribonucleoprotein, the cofactor for cholesterol biosynthetic genes (Sallam et al. 2016). Variation in the RALY gene (Fig. 2A) is associated with yellow versus black/brown pigmentation in mice and quail (Michaud et al. 1994; Nadeau et al. 2008), and is adjacent to two other pigmentation genes—ASIP (115,941 bp away) and EIF2S2 (30,223 bp away) in the reference genome (Figs. 2A and 4C). Hence, we refer to this region hereafter as the “ASIP‐RALY” gene block, and the specific SNP as the RALY SNP. Variation in the RALY SNP explains 60% of variation in crown (Figs. 3A and 3E), 57% of the variation in cheek (Figs. 3B and 3F), 49% variation in breast coloration (Figs. 3C and 3G), and 59% variation in flank streaking (Figs. 3D and 3H). The pure *occidentalis* population mostly contains GG homozygotes (referred to hereafter as OO; Fig. 3) at the RALY SNP, whereas the *townsendi* population contains CC homozygotes (referred to as TT).

Only two other SNPs were found to be significantly associated with our phenotypic traits. The first is on chr 4A at nucleotide location 5588235, inside an ortholog of the mammal Immunoglobulin Binding Protein 1 (IGBP1) gene, explaining 33% of the variation in the intensity of yellow on the breast (Fig. 2D). The second, explaining 7% of the variation in the extent of breast yellow (Fig. 2F), is on chr 1 at position 54673540, in the intergenic region between NOP2/Sun RNA Methyltransferase 3 (NSUN3) and Ephrin Type‐A Receptor A6 (EPHRA6). We did not detect SNPs that significantly explained variation in either bib darkening or green coloration on the back (Figs. 2G and 2H).

### INHERITANCE PATTERN

We estimated the dominance coefficient (*h*) of the O allele for each phenotype as

$$h=\left({\bar{Z}}_{TT}-{\bar{Z}}_{OT}\right)\;/\;\left({\bar{Z}}_{TT}-{\bar{Z}}_{OO}\right),$$

where $\bar{Z}$_OO_, $\bar{Z}$_OT_, and $\bar{Z}$_TT_ denotes the mean phenotype of OO, OT, and TT, respectively. To estimate confidence intervals, we conducted 10,000 iterations of bootstrap resampling of individuals, calculating the mean phenotypes and *h* each time, and thereby generating a bootstrap distribution of *h*. An *h* = 0.5 ($\bar{Z}$_OT_ = [$\bar{Z}$_TT_ + $\bar{Z}$_OO_]/2) would correspond to complete additivity, *h* = 0 to complete recessivity of the O allele ($\bar{Z}$_OT_ = $\bar{Z}$_TT_), whereas *h* = 1 to complete dominance of the O allele ($\bar{Z}$_OT_ = $\bar{Z}$_OO_). Partial recessivity of the O allele would correspond to 0 < *h* < 0.5, and partial dominance of the O allele to 0.5 < *h* < 1. The estimated *h* is 0.810 for crown darkening (95% CI, 0.682‐0.946), 0.908 for cheek darkening (95% CI, 0.745‐1), 0.557 for breast yellow intensity (95% CI, 0.332‐0.783), and 0.345 for flank streaking (95% CI, 0.174‐0.542).

### DIFFERENTIATION BETWEEN PARENTAL POPULATIONS

GWAS results are most informative when viewed in the context of genomic patterns of differentiation. There was low genome‐wide weighted average *F*_ST_ (Weir and Cockerham 1984) between allopatric *occidentalis* and *townsendi* populations (Weir and Cockerham's *F*_ST_ = 0.03). However, four high regions of differentiation (*F*_ST_ > 0.6) were found (Figs. 4A and S3; gene association and functions summarized in Table S4) that map to Zebra Finch chromosome (chr) 1A (nucleotide position 54442413, *F*_ST_ = 0.66), chr 5 (nucleotide position 25064223–25875302, mean *F*_ST_ = 0.75), chr 20 (at the RALY SNP, 1981369, *F*_ST_ = 0.90), and chr Z (66226657, *F*_ST_ = 0.82). SNPs in the vicinity of the RALY SNP did not show as strong a differentiation (Figs. 4A and 4B) in the GBS data, even though the minor allele frequencies were quite high (Table S3). The SNPs on chr 1 and chr 4A that were associated with breast coloration were not associated with *F*_ST_ peaks.

Such genomic architecture of differentiation revealed by GBS data is consistent with the pattern from the WGS data (Fig. 4B and 4C), which is based on a larger number of SNPs but a smaller number of individuals. The WGS data (genome‐wide average *F*_ST_ = 0.08; Fig. 4B red dotted line) are mostly consistent with the GBS data in showing low differentiation across much of the genome but a few strong narrow peaks at chr 1A, 4, 5, 20, and Z (Figs. 4B and S3). The *F*_ST_ peak on chr 20 is about 200‐kb wide (estimated from the WGS data in Fig. 4C) and includes the RALY SNP (Figs. 4A and 4C). In the WGS data (Figs. 4B and S3), which have much higher density of markers across the genome than the GBS dataset, this “island” of differentiation corresponds closely to the location of the ASIP‐RALY pigmentation gene block (Figs. 2A, 4B, and 4C). The RALY SNP revealed by the GBS data can be treated as a convenient marker representing this ASIP‐RALY differentiation gene block surveyed across a large number of individuals.

### EVIDENCE OF SELECTION ON THE ASIP‐RALY REGION

**Figure 5:**
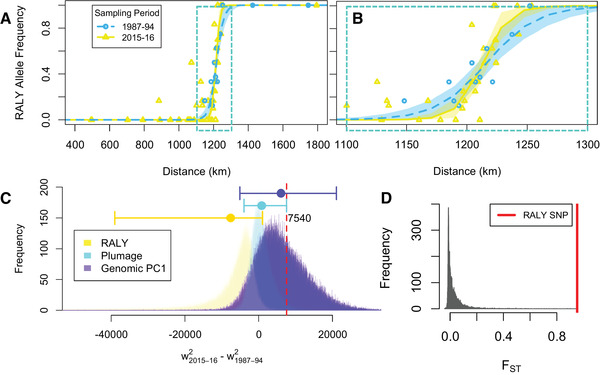
Evidence of selection on RALY or its linked region. The RALY SNP shows stable clines (sampled in 1987–1994 and 2015–2016) across the hybrid zone, which are extremely narrow (**A**; **B** is zoom in of A around 1100–1300 km) (**C**). **C**, The bootstrap distribution of *w* ^2^_2015‐2016_ – *w* ^2^_1987‐1994_ (the change of squared cline width between sampling periods) with RALY cline in yellow, plumage cline in cyan, and genomic PC1 cline in purple. Bootstrap mean and 95% CIs of *w* ^2^_2015‐2016_ – *w* ^2^_1987‐1994_ of are shown on the top of (**C**): the RALY cline (yellow) and plumage cline (cyan) are less than expected under neutral diffusion (7540 km^2^, red vertical line), whereas genomic cline (purple) is not. **D**, RALY is the extreme outlier in the distribution of *F*_ST_ between inland *townsendi* and *occidentalis* across the genome (RALY SNP *F*_ST_ value depicted by the red line).

Several lines of evidence indicate that the ASIP‐RALY region is under selection maintaining differentiation between the species, perhaps in part through causing low fitness of heterozygotes. First, it is the most extreme *F*_ST_ outlier (*F*_ST_ = 0.90) in the GBS data, whereas most of the genome is not very differentiated (genome‐wide *F*_ST_ = 0.03) (Fig. 5D). Second, the RALY SNP (representing the ASIP‐RALY gene block) demonstrated a narrow spatial cline that was stable in location over two decades (Fig. 5A). The RALY cline is narrower in width (*w* = 42.21 km; 95% CI, 22.44‐62.18) than the genomic cline (*w* = 112.28 km; 95% CI, 73.45‐151.10) in 2015–2016 (Table S5). The RALY cline center was at 1216.35 km in 1987–1994 (Fig. 5A, blue curve; Table S5), and did not significantly shift from that in the 2015–2016 sampling (Fig. 5A, yellow curve; Table S5), occurring at 1213.14 km. The genomic cline and the plumage cline stayed stable in position as well (Wang et al. 2019).

Furthermore, the increase of the width of the RALY cline was less (95% CI of *w* ^2^_2015‐2016_ – *w* ^2^_1987‐1994_: –38,573.70 to 1179.23 km^2^) than the plumage cline (95% CI of *w* ^2^_2015‐2016_ – *w* ^2^_1987‐1994_: –3978.35 to 7536.59 km^2^) (Wang et al. 2019), which increased less than expected under neutral diffusion (*w* ^2^_2015‐2016_ – *w* ^2^_1987‐1994_ = 7540 km^2^) (Fig. 5C; see Wang et al. 2019) for explanation of method). The whole‐genome cline did not deviate significantly from neutral diffusion (95% CI of *w* ^2^_2015‐2016_ – *w* ^2^_1987‐1994_: –5125.63 to 21,056.13 km^2^) (Wang et al. 2019). No significant narrowing or widening of the RALY cline, plumage cline, nor genomic cline was observed (0 was included in the 95% CIs of *w* ^2^_2015‐2016_ – *w* ^2^_1987‐1994_ above).

## Discussion

Hybrid zones have long been considered “laboratories for the study of speciation” (Hewitt 1988; Barton and Hewitt 1989). For many decades, they have provided opportunities to examine the association between various traits and reproductive isolation (Slatkin 1973; Endler 1977; Liou and Price 1994; Gompert et al. 2012; Shurtliff 2013). With recent advances in sequencing technology, they now provide an opportunity to uncover the genomic regions underlying traits distinguishing species and the role of those regions in genome‐wide differentiation of species. The present study provides a clear example: a narrow genomic region encompassing just three genes is highly associated with at least four phenotypic traits and this region is among the most differentiated parts of the genome, displaying a narrow and stable cline consistent with selection against hybrids due to this genomic region. If one asks, “What makes *Setophaga townsendi* and *S. occidentalis* different species?” a big part of the answer must involve the ASIP‐RALY gene block. Below, we discuss the evidence in support of this conclusion.

### GENETIC MECHANISM OF COLOR DIFFERENTIATION

Variation in the RALY SNP explains (in a statistical sense) up to 60% of the variation in four out of seven plumage traits that differ between our two study species. A low‐resolution sequencing approach such as GBS, which sequences less than 1% of the genome, would be unlikely to detect a SNP that is directly mechanistically causing variation in the phenotype. It is more likely that the RALY SNP is closely linked to one or more directly causal DNA variants (SNPs or other variants) affecting phenotypic differences. It is also possible that multiple linked SNPs in this region are responsible for the variation in different phenotypic traits, such that the apparent pleiotropic effect (i.e., strong association with three plumage traits) of the RALY SNP might be due to separate causal genes that are closely linked. For these reasons, we consider the three genes in the same narrow differentiation peak as the RALY SNP, as well as regulatory sequences near these genes, to be candidates for the specific sites that directly influence the phenotypes.

Each of the three genes in the ASIP‐RALY island of differentiation has been invoked as being associated with color in other species. EIF2S2 has been associated with human skin pigmentation (Liu et al. 2015). ASIP, which codes for agouti signaling protein, is well known for its influence on skin pigment in vertebrates by binding to melanocortin receptors and competitively excluding its agonists, preventing black/brown pigmentation (Lovett et al. 1987; Horrell et al. 2016). The RALY gene encodes for heterogeneous nuclear ribonucleoprotein, the RNA binding protein that is known to regulate the expression of its downstream gene ASIP (Nadeau et al. 2008). Deletion of RALY in Japanese quails and mice leads to novel transcripts of ASIP, which cause skin phenotype transition from brown/black to yellow (called “lethal yellow”) (Michaud et al. 1994; Nadeau et al. 2008). In mice, a lethal yellow mutation (Ay) in ASIP exhibits effects on skin coloration that are regulated by the 5’ regulatory region flanking ASIP (Michaud et al. 1994). Genetic variants of RALY in warblers could result in differential expression of ASIP leading to variations in carotenoid and melanin patterning.

In birds, ASIP has been involved in genomic divergence between closely related species in *Vermivora* (Toews et al. 2016b), *Sporophila* (Campagna et al. 2017), and *Lonchura* (Stryjewski and Sorenson 2017). Our finding of high differentiation in the RALY SNP in this *Setophaga* system and high association with plumage in the hybrid zone is consistent with inferences from these comparative genomic studies. Future studies should investigate the functional interaction of RALY and ASIP in plumage differentiation, and whether there is any convergent mutation in ASIP between *Vermivora* and *Setophaga*.

A previous genome‐wide association study in a hybrid zone between *Setophaga coronata auduboni* and *S. c. coronata* revealed a genomic region containing the candidate gene SCARF2 on chromosome 15 to be associated with multiple carotenoid and melanin traits (Brelsford et al. 2017). In contrast, we found little differentiation (WGS, *F*_ST_ ∼ 0.07) in SCARF2 in our study of a species pair of the same genus. Instead, we found that the ASIP‐RALY gene block on chr 20 shows the main association with both carotenoid and melanin pigment patches.

On top of the strong ASIP‐RALY association with various plumage traits, there are very likely other regions of the genome contributing to further refinement of species‐specific coloration in this system. Specifically, other SNPs on chr 4A and chr 1 are significantly associated with intensity and extent of breast coloration. In addition, there might be narrow but important genetic regions that this GBS dataset did not cover. However, the GBS and WGS datasets produced broadly similar overall genomic differentiation landscapes. That, together with the finding of strong associations between some GBS‐identified SNPs and plumage, gives us confidence in using GBS‐based GWAS to reveal the key regions underlying traits differentiating species.

### INHERITANCE OF COLOR PHENOTYPES

Previous studies in the lab (Michaud et al. 1994; Nadeau et al. 2008) or natural populations (Ryan et al. 2018) converged in inferring an inheritance pattern of ASIP‐RALY in which the melanic form is the recessive phenotype. Consistent with this pattern and the prediction made by previous study in this system (Rohwer and Wood 1998), we found a pattern of almost complete dominance of the O allele in cheek darkening (*h* = 0.908). Moreover, we observed partial dominance of the O allele (with dominance coefficient ranging from 0.5345 to 0.810; Fig. 3) in three of the four phenotypes that were significantly associated with the RALY locus. Interestingly, our data suggest the opposite pattern for RALY association with flank streaking, where the O allele is likely to be partially recessive (*h* = 0.345, although the confidence interval includes *h* = 0.5). Such “opposing genetic dominance” (Ohshima 2008, 2010) of the same locus regulating different plumage traits could result in mismatched plumage ancestry in heterozygotes, which might disrupt hybrid plumage signaling and reduce hybrid fitness (Irwin 2020).

The observation of four distinct plumage features being strongly associated with variation in the RALY SNP suggests pleiotropy, in which variation in a single gene affects multiple traits. Another possibility is that different but closely physically linked genes are affecting different traits, with each gene affecting one or more of pigmentation traits. In this case, it is possible that ASIP, EIF2S2, and RALY essentially function as a “supergene” (a set of closely linked and functionally related genes) affecting a suite of pigmentation traits. Segregating supergenes can underpin differentiation of a set of traits, facilitating divergence among closely related species (Joron et al. 1999; Steiner et al. 2007; Lowry et al. 2009; Renaut et al. 2013; Jay et al. 2018).

Previous laboratory study of RALY mutants revealed pleiotropic effects on other traits in addition to skin coloration, including obesity and diabetic condition (Michaud et al. 1994), which sheds light on the potential RALY pleiotropy in this system as well. Distinguishing between pleiotropic effects of a single gene versus combined effects of multiple closely linked genes is beyond scope of the present study. Either way, the consequences for the population genomics of speciation and hybridization are similar, as reduced gene flow in that one small chromosomal region can maintain multiple phenotypic differences between the species.

### SELECTION MAINTAINING DIFFERENCES BETWEEN SPECIES

In addition to the potential pleiotropic effect on coloration, the RALY SNP is associated with selection maintaining differences between the species. We observed almost fixed ASIP‐RALY differences between the parental populations (Fig. 4) as well as a stable and narrow RALY cline (Fig. 5), inconsistent with neutral diffusion and suggesting that heterozygous combinations cause reduced fitness. Although the mechanism by which ASIP‐RALY is associated with reproductive isolation is yet to be discovered, here we suggest two possibilities that can be tested in future studies. First, ASIP‐RALY could facilitate assortative mating. Melanin and carotenoid plumage traits often influence mate choice (Kingma et al. 2008; McGraw 2008). Given that this gene block is associated with a suite of interspecific plumage differences, future study should test if these plumage differences are employed as mating signals, and whether this gene block is associated with mate preferences. However, a recent simulation study showed that incomplete assortative mating is ineffective, on its own, at maintaining stable clines across hybrid zones (Irwin 2020). Alternatively, the ASIP‐RALY gene block could be associated with low fitness of hybrids. The plumage differences underpinned by ASIP‐RALY were shown to be involved in male‐male competition (Pearson et al. 2000; Townsend et al. ; Owen‐Ashley and Butler 2004), in which males with *townsendi*‐like plumage were socially dominant over *occidentalis*‐like plumage. Because our results suggest that T alleles of the RALY SNP may show reverse dominance for different trait (discussed above), heterozygotes of the RALY SNP may demonstrate patchy plumage ancestries leading to signal interference and disadvantage at territorial combat. Future study should test in more detail the role of these plumage signals in mate preference and social competition within the hybrid zone. Such further investigation would be important, in that genetic evidence for pleiotropic loci that influence both selected traits and reproductive isolation has been scarce (see review by Nosil 2012). Among the limited existing examples, the classic cases involve coloration: the wingless gene affecting reproductive isolation and wing coloration in *Heliconius* butterflies (Kronforst et al. 2006), and the YUP locus that affects pollinator isolation and flower coloration in monkey flowers (Bradshaw and Schemske 2003).

### GENOMIC ARCHITECTURE OF SPECIATION

Neutral or weakly selected genomic differentiation accumulated in allopatry can be homogenized by gene flow at secondary contact, yet genetic regions that are differentially selected can be maintained. In this system, genomic differentiation built up over the course of isolation (due to Pleistocene glaciations; Weir and Schluter 2004; Krosby and Rohwer 2009) can result in genomic incompatibilities that lower hybrid fitness and prevent merging of the species. In addition, new genomic barriers can arise after secondary contact in response to selection for reproductive isolation, extending the genomic barrier against gene flow (Hartl 1977; Felsenstein 1981; Barton 1983).

The ASIP‐RALY gene block stands out as a prominent differentiated region that is resistant to gene flow between the species and underlies variation in multiple plumage patches, whereas most of the genome is similar between the species, reflected as the lack of differentiation in allopatry and/or homogenization across the hybrid zone. This suite of melanin‐ and carotenoid‐related plumage patches (crown, cheek, breast coloration, and flank streaking) differs diagnostically between these species and is likely under some form of selection (discussed above) that maintains the stable and narrow hybrid zone at the species boundary (Wang et al. 2019). The fact that both the cline width and the change in cline width are smaller for the RALY SNP than the rest of the genome (Fig. 5) supports this idea.

### SPECIATION FUTURE

The similarity between *townsendi* and *occidentalis* in the great majority of the genome raises a question regarding the distinctiveness of these two taxa and their future. The two forms have noticeably different appearances, explaining why they have been treated as two species ever since their initial description. Clear genomic differentiation, however, occurs in only a few small chromosomal regions, and the extensive hybrid zone between the species complicates their treatment as distinct species. Furthermore, there is substantial evidence that *townsendi* along the coast of British Columbia and Alaska resulted from ancient hybridization between *occidentalis* and *townsendi* (Krosby and Rohwer 2009), raising questions as to their distinctness as evolutionarily independent forms. Despite this, the present hybrid zone between birds that have the appearance of *townsendi* and those with the appearance of *occidentalis* has been narrow over decades (∼12 generations), suggesting stability of the two forms and substantial independence going forward. This is particularly true for the ASIP‐RALY gene region and perhaps those few other genomic peaks of differentiation (Fig. 4). A key outstanding question is whether these regions of differentiation and the current dynamics of the hybrid zone will allow further differentiation to build up, perhaps due to the process of reinforcement (Liou and Price 1994; Servedio and Noor 2003) in which low fitness of hybrids leads to the evolution of stronger assortative mating.

## AUTHOR CONTRIBUTIONS

SW and DI co‐designed the study with help from SR. SW conducted 2015–2016 field sampling, lab work, and genomic analysis with advice from DI. DRdZ designed the plumage pigment quantification protocol and JM conducted all the plumage pigmentation measurement. DPLT and IJL provided WGS data; DPLT guided SW for WGS data analysis. SW completed the first draft, which was edited back‐and‐forth by DI and SW, before other authors commenting and editing toward the final version.

## DATA ARCHIVING

All the sequence data in this study are provided on SRA (accession number: PRJNA573930; ID: 573930). The metadata sheets involved in the analysis and figure production are deposited (https://doi.org/10.5061/dryad.bnzs7h470).

Associate Editor: Z. Gompert

## Supporting information


**Fig. S1** Field photos of a hybrid male showing three different angles: 1) frontal with head tilted up showing throat badge and breast measurements), 2) profile showing the cheek, and 3) from above showing the crown.
**Fig. S2** Pairwise scatterplots among 7 plumage variables (crown, cheek, bib coloration, extent and intensity of breast yellow, back coloration, and flank streaking) in the GWAS.
**Fig. S3** Weir and Cockerham *F_ST_* scan of the WGS data, in which each dot represents a 10kb non‐overlapping window (**A**), where peaks were found on chromosome 1A (**B**), 4 (**C**), 5 (**D**), 20 (**E**), and Z (**F**).
**Table S1** Sample size (the number of individuals with GBS data and at least one plumage variable quantified) involved in the GWAS for each sampling period and population range (parental zones and the hybrid zone).
**Table S2** Sample size of the 7 plumage variables (diagonal, bolded) in which the intersect of the pairwise plumage variables were shown in off diagonal.
**Table S3** Allele frequencies of SNPs between RALY and ASIP in hybrid zone and parental zones based on the GBS dataset.
**Table S4** SNPs (in the GBS dataset) with *F_ST_* > 0.6 and their position, association to genes and molecular functions.
**Table S5** Center and width of the RALY cline, plumage cline, and genomic cline of historical (1987‐94) versus recent (2015‐16) sampling.
