# Supplementary material for: Selection on a small genomic region underpins differentiation in multiple color traits between two warbler species
Source: Evol Lett. 2020 Oct 19;4(6):502–15. doi: 10.1002/evl3.198 (PMC7719548; doi:10.1002/evl3.198)
Supplement: Supplementary file 1 — Fig. S1 Field photos of a hybrid male showing three different angles: 1) frontal with head tilted up showing throat badge and breast measurements), 2) profile showing the cheek, and 3) from above showing the crown. Fig. S2 Pairwise scatterplots among 7 plumage variables (crown, cheek, bib coloration, extent and intensity of breast yellow, back coloration, and flank streaking) in the GWAS. Fig. S3 Weir and Cockerham FST scan of the WGS data, in which each dot represents a 10kb non‐overlapping window (A), where peaks were found on chromosome 1A (B), 4 (C), 5 (D), 20 (E), and Z (F). Table S1 Sample size (the number of individuals with GBS data and at least one plumage variable quantified) involved in the GWAS for each sampling period and population range (parental zones and the hybrid zone). Table S2 Sample size of the 7 plumage variables (diagonal, bolded) in which the intersect of the pairwise plumage variables were shown in off diagonal. Table S3 Allele frequencies of SNPs between RALY and ASIP in hybrid zone and parental zones based on the GBS dataset. Table S4 SNPs (in the GBS dataset) with FST > 0.6 and their position, association to genes and molecular functions. Table S5 Center and width of the RALY cline, plumage cline, and genomic cline of historical (1987‐94) versus recent (2015‐16) sampling. [file EVL3-4-502-s001.docx]

**Supplementary Information**


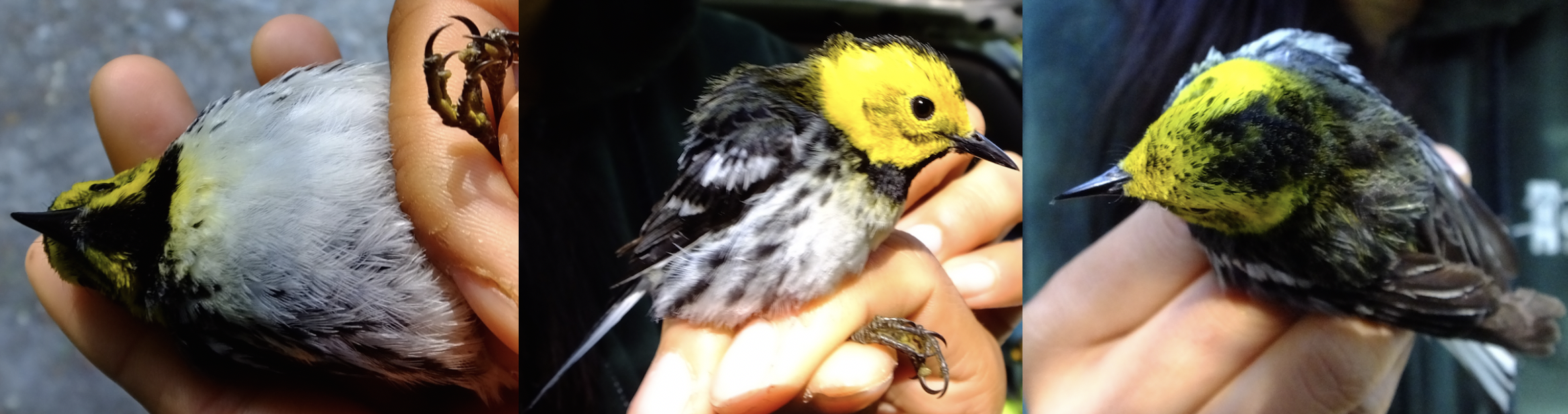


**Fig. S1** Field photos of a hybrid male showing three different angles: 1) frontal with head tilted up showing throat badge and breast measurements), 2) profile showing the cheek, and 3) from above showing the crown.

**Fig. S2** Pairwise scatterplots among 7 plumage variables (crown, cheek, bib coloration, extent and intensity of breast yellow, back coloration, and flank streaking) in the GWAS. All the pairwise correlations are significant (Pearson product-moment correlation test with Bonferroni correction, *p* < 0.05), except between bib.L (bib coloration) and breast.yellow (the extent breast yellow), bib.L and breast.b (the intensity of breast yellow), bib.L and back.a, bib.L and crown.b (crown coloration).


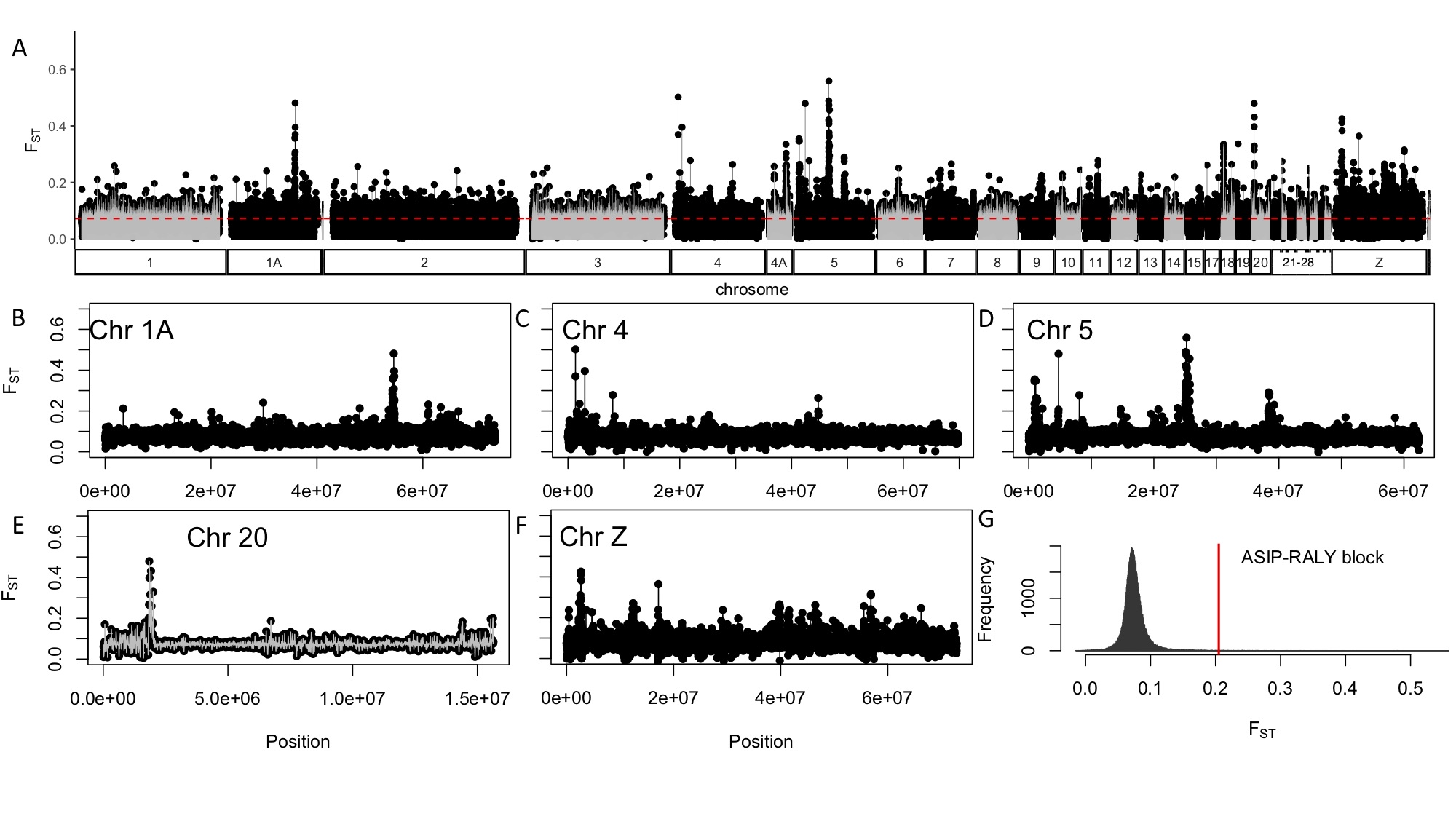


**Fig. S3** Weir & Cockerham *F_ST_* scan of the WGS data, in which each dot represents a 10kb non-overlapping window (**A**), where peaks were found on chromosome 1A (**B**), 4 (**C**), 5 (**D**), 20 (**E**), and Z (**F**). **G**, the ASIP-RALY gene block (mean *F_ST_* in the ~230kb block) demonstrates extremely high *F_ST_* relative to the rest of the genome.

**Table S1** Sample size (the number of individuals with GBS data and at least one plumage variable quantified) involved in the GWAS for each sampling period and population range (parental zones and the hybrid zone). These include and 213 from the Cascade hybrid zone in Washington, U.S.A, 45 *occidentalis* captured in Southern Oregon or California, U.S.A., and 7 *townsendi*, captured in Northeast Washington, Idaho, or Montana, U.S.A.

| Sampling zone |  | Sampling period | |
| --- | --- | --- | --- |
|  | 1987-94 | 2005-2008 | 2015-16 |
| *S. occidentalis* | 0 | 0 | 45 |
| hybrid zone | 41 | 15 | 157 |
| *S. townsendi* | 0 | 0 | 7 |

**Table S2** Sample size of the 7 plumage variables (diagonal, bolded) in which the intersect of the pairwise plumage variables were shown in off diagonal.

|  | Crown.B | Cheek.B | Bib.L | Breast.B | Breast.Y.Ext | Flank.Streaking | Back.A |
| --- | --- | --- | --- | --- | --- | --- | --- |
| Crown.B | **229** | 228 | 227 | 229 | 224 | 155 | 228 |
| Cheek.B | 228 | **237** | 235 | 237 | 232 | 162 | 236 |
| Bib.B | 227 | 235 | **237** | 237 | 233 | 164 | 236 |
| Breast.B | 229 | 237 | 237 | **239** | 234 | 164 | 238 |
| Breast.Y.Ext | 224 | 232 | 233 | 234 | **235** | 161 | 233 |
| Flank.Streaking | 155 | 162 | 164 | 164 | 161 | **189** | 163 |
| Back.A | 228 | 236 | 236 | 238 | 233 | 163 | **238** |

**Table S3** Allele frequencies of SNPs between RALY and ASIP in hybrid zone and parental zones based on the GBS dataset. The causal genetic mechanism for plumage coloration might be narrower than the ASIP-RALY linkage block around the RALY SNP. The RALY SNP is in physical proximity to the two other pigmentation genes mentioned above, although 3 other SNPs (1955244, 1972476, and 1972481) that are 8888-26125 bp away from RALY SNP (Fig. 2 A), physically closer to ASIP and EIF2S2, did not show association with phenotype (Fig. 2 A). Two of these SNPs showed low minor allele frequencies (0.05-0.09) in the hybrid zone, and thus are not expected to be highly associated with trait variation. However, the fact that SNP 1972476 (closer to the other genes than the candidate RALY SNP) demonstrated similar minor allele frequency as the significant RALY SNP and was not significantly associated with plumage coloration highlights the importance of the region around position 1981369 inside the RALY gene. Either way, the RALY SNP represents the ASIP-RALY gene block and appears to have an effect on multiple species-diagnostic coloration traits.

|  | **POS** | **allele1:freq** | **allele2:freq** |
| --- | --- | --- | --- |
| Hybrid zone | |  |  |
|  | 1955244 | T:0.946 | A:0.054 |
|  | 1972476 | A:0.72 | C:0.28 |
|  | 1972481 | T:0.917 | C:0.083 |
|  | 1981369 | C:0.375 | G:0.625 |
| *townsendi* zone | |  |  |
|  | 1955244 | T:1 | A:0 |
|  | 1972476 | A:0.5 | C:0.5 |
|  | 1972481 | T:0.656 | C:0.344 |
|  | 1981369 | C:1 | G:0 |
| *occidentalis* zone | |  |  |
|  | 1955244 | T:0.95 | A:0.05 |
|  | 1972476 | A:0.944 | C:0.056 |
|  | 1972481 | T:0.972 | C:0.028 |
|  | 1981369 | C:0.048 | G:0.952 |

**Table S4** SNPs (in the GBS dataset) with *F_ST_* > 0.6 and their position, association to genes and molecular functions.

| Chromosome | Position | *F_ST_* | Relation to genes | Gene(s) | Function |
| --- | --- | --- | --- | --- | --- |
| 1A | 54442413 | 0.656 | intergenic | GRM8, ENSTGUG00000004218 | G protein-coupled receptor activity, glutamate receptor activity, telomere maintenance, DNA binding |
| 5 | 25064223 | 0.822 | intron | UBR1 | ubiquitin-protein transferase activity |
| 5 | 25174918 | 0.855 | coding | ENSTGUG00000011205 | DNA binding |
| 5 | 25746680 | 0.644 | intergenic | DPF3, RGS6 | histone acetyltransferase activity; G protein-coupled receptor signaling pathway, intracellular signal transduction |
| 5 | 25783847 | 0.772 |  |  |  |
| 5 | 25875302 | 0.649 | intron | RGS6 | G protein-coupled receptor signaling pathway, intracellular signal transduction |
| 20 | 1981369 | 0.892 | intron | RALY | DNA and RNA binding, cholesterol biosynthesis |
| Z | 66226657 | 0.818 | intron | BBOX1, TNPO1 | carnitine biosynthesis, oxidation-reduction; protein import into nucleus, intracellular protein transport |

**Table S5** Center and width of the RALY cline, plumage cline, and genomic cline of historical (1987-94) versus recent (2015-16) sampling.

|  | RALY cline | | Plumage Cline | | Genomic Cline | |
| --- | --- | --- | --- | --- | --- | --- |
| Period | 1987-94 | 2015-16 | 1987-94 | 2015-16 | 1987-94 | 2015-16 |
| Center | 1216.35 | 1213.14 | 1207.93 | 1207.65 | 1218.84 | 1223.28 |
| 95%CI (Center) | (1,208.61, 1,224.09) | (1,208.89, 1,217.39) | (1,204.92, 1,210.93) | (1,203.48, 1,211.83) | (1,214.48, 1,223.20) | (1,216.88, 1,229.68) |
| Width | 82.64 | 42.31 | 64.90 | 62.06 | 94.35 | 112.28 |
| 95%CI (Width) | (44.11, 121.17) | (22.44, 62.18) | (50.77, 79.03) | (42.703, 81.42) | (72.11, 116.58) | (73.45, 151.10) |

**Supplementary Methods**

*GBS pipeline*

Following Alcaide et al. (2014), we prepared genotyping-by-sequencing (GBS; (Elshire et al. 2011)) libraries from 352 individual DNA samples. In brief, genomes were digested with restriction enzyme and ligated with barcode and adaptors, amplified with PCR, and size selected (fragment length of 300 - 400 bp) for sequencing. Libraries were sequenced at Genome Quebec with paired-end sequencing (read length = 125 bp) on an Illumina HiSeq 2500 automated sequencer. The resulting sequences were processed following the pipeline of Irwin et al. (2016). We demultiplexed the reads with a custom script and trimmed them using Trimmomatic 0.36 (Bolger et al 2014) [TRAILING:3 SLIDINGWINDOW:4:10 MINLEN:30], then we aligned reads to a *Taeniopygia guttata* reference version 3.2.4 (Warren et al. 2010) using bwa (Li et al 2009) (default settings). We assumed synteny of *Setophaga* and *T. guttata* genomes based on evolutionary stasis of rearrangement in avian genomes (Ellegren 2010; Zhang et al. 2014), but the conclusions of this study would be unlikely to be affected by a moderate number of rearrangements. However, if there is rearrangement within ASIP-RALY gene block, future study should consider additional representative SNPs within this gene block. We conducted SNP calling with GATK (McKenna et al. 2010), which produced 4,097,089 SNPs. The SNP filtering was done with VCFtools 0.1.14 (Danecek et al. 2011), which includes removing indels, requiring genotype quality (GQ) > 20, minor allele frequency (MAF) $\geq$ 0.05, removing loci with >30% missing data, and only including biallelic SNPs, resulting in 21,852 SNPs remaining.

*Color-correction controlling for age effect*

Plumage darkness was found to be associated with age-classes with the younger birds darker than the older ones (Jackson et al. 1992). To control for this effect we followed the age-correction protocol of our previous study (Wang et al. 2019). Briefly, we aged each bird as our previous study (Wang et al. 2019) and transformed each of the 7 plumage variable by the corresponding correction factor. The correction factor is determined by the mean difference of the old and young bird within each quartile of each trait distribution.

*Color-correction for museum specimens*

In contrast to the plumage coloration of live birds, carotenoid and melanic colors could decay in the specimens over museum preservation. There is indeed slightly less coloration in the museum specimens than live bird of similar genomic backgrounds (for crown: F = 23.38, *p* < 10^-5^; for cheek: F = 108.63, *p* < 10^-15^; for bib coloration, F = 101.48, *p* < 10^-15^; for extent of breast yellow: F = 14.98, *p* = 0.0001; for intensity of breast yellow: F = 73.43, *p* < 10^-14^; for flank streaking: F = 51.40, *p* < 10^-11^; but not for back coloration: F = 2.06, *p* = 0.15). To control for such effects, we corrected museum color measurements with the color measurements from live birds of similar genomic background, represented by genomic eigenvector 1 (EV1). Genomic EV1 was calculated with the 21,852 SNPs with SNPRelate (Zheng et al. 2012). For each quantile of genomic EV1 of the museum specimens (corresponding to a set of specimens), the live birds with genomic EV1 within this quantile is used as the ‘field reference set’. The difference in the mean plumage score of the field reference set and the specimens set becomes the ‘correction factor’, which was added to the plumage score of the museum specimens set. This museum preservation effect correction step was performed after the age-correction (see Methods).

*Geographical cline analysis on candidate loci*

As one SNP within the RALY gene (see Results) stood out in the above analysis as particularly strongly associated with plumage variation, we investigated the spatial and temporal variation in this locus relative to the plumage hybrid index (HI) and rest of the genome following Wang et al. (2019), which was respectively based on the scores of the 8 plumage scores (with 0 representing pure *occidentalis* and 1 being pure *townsendi*), and the scaled genomic PC1 (with 0 representing pure *occidentalis* and 1 being pure *townsendi*). The HI calculated with the current plumage quantification is highly correlated with the plumage scores in our previous study (r) = 0.83, *p* < 2.2e-16**)**. We selected the same set of individuals from which the genomic clines (Wang et al. 2019) were calculated, and fit the relationship between allele frequency of the RALY SNP and location using an equilibrium geographic cline model (Szymura and Barton 1986; Gay et al. 2008).

Geographical cline analysis was done following Wang et al. (2019). Briefly, we collapsed the two-dimensional sampling into a one-dimensional transect by measuring the location of each site to the 0.5 isocline of the plumage HI in the historical sampling (Rohwer and Wood 1998), as follows. First, a Local Polynomial Regression Fitting (LOESS) model in R (R Core team 2015) was used to fit variation in HI across the hybrid zone, and this model was used to estimate the HI = 0.5 isocline in the 1987-94 sampling (Rohwer and Wood 1998). Then for each site, the shortest distance to the 0.5 isocline was calculated with the *sp* package (Pebesma and Bivand 2005). The sites east or west of the isocline were specified as having positive versus negative distance values, respectively. We added 1200 km to the distance score of each site so that all distance values are above zero, while the relative distance of each site to the isocline is preserved. Then the data was fit to the equilibrium sigmoidal cline model (Szymura and Barton 1986; Gay et al. 2008) $y= \frac{1}{{1+e}^{-\frac{4(x-c)}{w}}}$, in which cline center (*c*) and width (*w*) was estimated (where *y* is HI and *x* is location with respect to the HI = 0.5 isocline). To examine whether selection (i.e., divergent selection and/or selection against hybrids) is acting on the candidate loci, we followed Wang et al. (2019) and tested whether the widening of cline width (*w^2^_2015-16_* -*w^2^_1987-94_*) is significantly less than expected under the neutral diffusion model (Barton and Hewitt 1985). To understand the RALY cline width change relative to the plumage and genomic cline, we compared *w^2^_2015-16_* -*w^2^_1987-94_* relative to the plumage and genomic HI cline Wang et al. (2019).

Supplementary Results

*SCARF2*

Previous admixture mapping in the same genus, *Setophaga coronata* *auduboni*/*S. c. coronata* revealed a genomic region containing the candidate gene SCARF2 (position 295,917-316,526) on chromosome 15 to be associated with multiple carotenoid and melanin traits (Brelsford et al. 2017). We examined this genetic region and found little differentiation (WGS dataset, *F_ST_* ~ 0.07) in SCARF2 in this *occidentalis* X *townsendi* hybrid zone. The GBS data did not cover the SCARF2 gene, but contains its flanking region, at position 267,260 and 340,616. We also observed little differentiation at these sites (*F_ST_* = 0.15, 0.014). There is no association of these two sites with the plumage variations (*p* > 0.05).
